# An updated explanation of ancestral karyotype changes and reconstruction of evolutionary trajectories to form *Camelina sativa* chromosomes

**DOI:** 10.1186/s12864-020-07081-0

**Published:** 2020-10-12

**Authors:** Zhikang Zhang, Fanbo Meng, Pengchuan Sun, Jiaqing Yuan, Ke Gong, Chao Liu, Weijie Wang, Xiyin Wang

**Affiliations:** 1 School of Life Sciences, North China University of Science and Technology, Tangshan, 063210 Hebei China; 2 College of Pharmacy, Chengdu University of Traditional Chinese Medicine, Chengdu, 610075 China; 3 Institute for Genomics and Bio-Big-Data, Chengdu University of Traditional Chinese Medicine, Chengdu, 610075 China

**Keywords:** Brassicaceae, *C. sativa*, Chromosome, Karyotype, Polyploid

## Abstract

**Background:**

Belonging to lineage I of Brassicaceae, *Camelina sativa* is formed by two hybridizations of three species (three sub-genomes). The three sub-genomes were diverged from a common ancestor, likely derived from lineage I (Ancestral Crucifer karyotype, ACK). The karyotype evolutionary trajectories of the *C. sativa* chromosomes are currently unknown. Here, we managed to adopt a telomere-centric theory proposed previously to explain the karyotype evolution in *C. sativa*.

**Results:**

By characterizing the homology between *A. lyrata* and *C. sativa* chromosomes, we inferred ancestral diploid karyotype of *C. sativa* (ADK), including 7 ancestral chromosomes, and reconstructed the evolutionary trajectories leading to the formation of extant *C. sativa* genome. The process involved 2 chromosome fusions. We found that sub-genomes Cs-G1 and Cs-G2 may share a closer common ancestor than Cs-G3. Together with other lines of evidence from Arabidopsis, we propose that the Brassicaceae plants, even the eudicots, follow a chromosome fusion mechanism favoring end-end joining of different chromosomes, rather than a mechanism favoring the formation circular chromosomes and nested chromosome fusion preferred by the monocots.

**Conclusions:**

The present work will contribute to understanding the formation of *C. sativa* chromosomes, providing insight into Brassicaceae karyotype evolution.

## Background

Brassicaceae (mustard family) is one of the largest groups in plants, being composed of an approximate 3709 species, classified into 338 genera [1]. It includes several species of prominent scientific and economic importance. According to phylogenetic relationship, Camelineae species (*Arabidopsis thaliana*, *Arabidopsis lyrata,* and *Capsella rubella*) and Brassica species (*Brassica rapa*, *Brassica nigra* and *Brassica oleracea*) respectively represent lineage I and lineage II, two of three well-supported lineages among the Brassicaceae [2, 3].

**Fig. 1:**
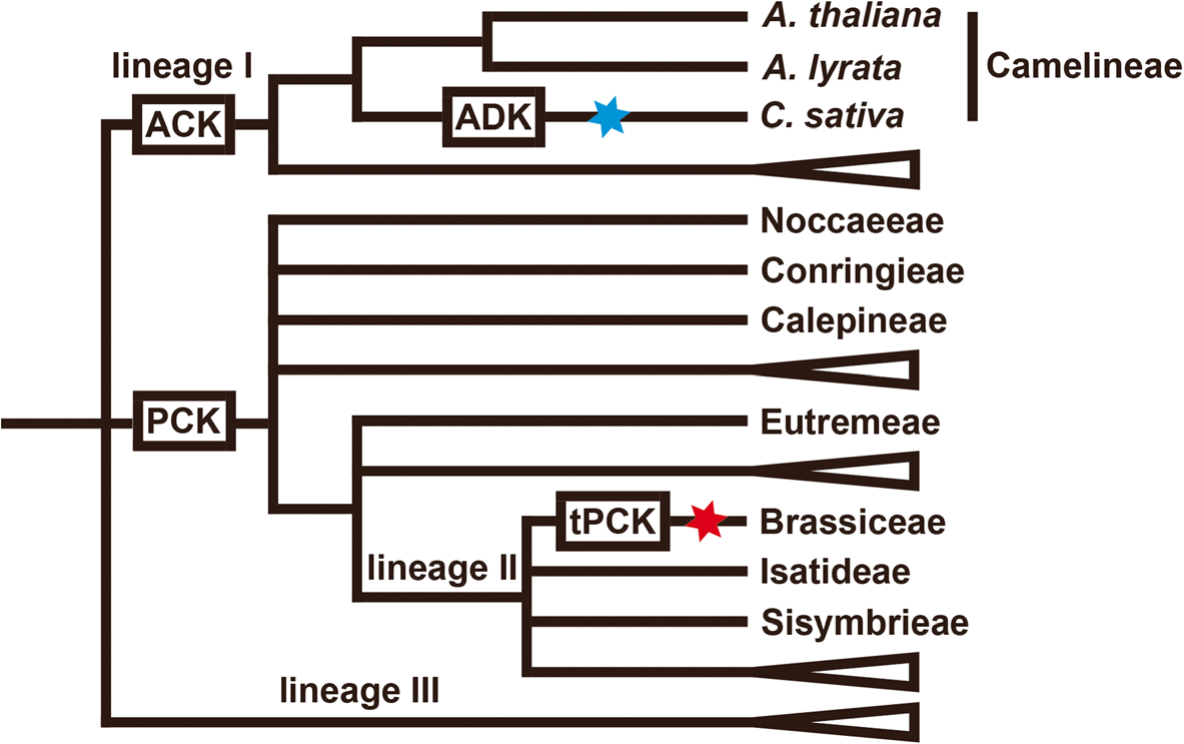
Phylogenetic relationship of selected tribes and species within the Brassicaceae. The red star refers to the Brassiceae-specific whole-genome triplication and blue star refers to a whole-genome triplication event relative to the crucifer model *A. thaliana*. Adopted and modified from Mandáková and Lysak (2008), Franzke et al. (2011) and Cheng et al. (2013)

With the rapid increase of Brassicaceae genome assemblies, reconstructing ancestral genome can help understand the evolutionary history of the extant Brassicaceae families and species. With genetic maps of *A. lyrata* and *C. rubella*, Schranz et al. defined 24 conversed GBs (labelled as A-X) related to ancestral karyotype (AK, *n* = 8) [4]. Ancestral crucifer karyotype (ACK, n = 8), improved from AK, is recognized as ancestral state of lineage I (Fig. 1 and 4a), based on the fact that most base common number of chromosomes is eight [5]. Besides, they reconstructed Proto-Calepineae karyotype (PCK, *n* = 7) as ancestral karyotype of 6 Brassicaceae tribes (Fig. 1). While PCK is inherited in three of the six tribes (Calepineae, Conringieae, and Noccaeeae), which belongs to lineage II, the rest three tribes (Eutremeae, Isatideae, and Sisymbrieae) is characterized by an additional translocation comparing to PCK, which is referred as translocation Proto-Calepineae Karyotype (tPCK, *n* = 7). Cheng et al. provided evidence that tPCK represents ancestral karyotype of the mesohexaploid *B. rapa*, the genus Brassica, and the tribe Brassiceae, by comparing three ancestral sub-genomes of Chinese cabbage (*B. rapa*) with PCK and tPCK [6].

Running through the evolutionary history of plant kingdom, polyploidization continually led to genome doubling/tripling, genome repatterning, and gene loss, characterizing genome instability and fractionation [7–9]. Interestingly, chromosome numbers could be much reduced to a kind of normal range after rounds of polyploidization. After two extra Brassicaceae-common duplications (BCD) [10], *A. thaliana* has only five base chromosomes. It was proposed that chromosome number reduction (CNR) was often the result of reciprocal translocations, which combined two chromosomes into a larger one and a smaller one, and the smaller chromosome got lost during meiosis [11]. For example, ACK and PCK shared the same karyotype of five chromosomes (AK1–4, and 7), AK6/8 and AK5/6/8 in PCK formed by reciprocal translocations between AK5, AK6 and AK8, resulting in chromosome number reduction from eight to seven [5]. Notably, an alternative telomere-centric model suggests that the removal of telomeres caused chromosome fusion and chromosome number reduction during the karyotype evolution, and explained the molecular dynamics of chromosome reformation [12]. Based on the telomere-centric model, ancestral karyotypes and evolutionary trajectories of chromosomes were reconstructed for Arabidopsis, grasses, and legumes [12–14].

**Fig. 2:**
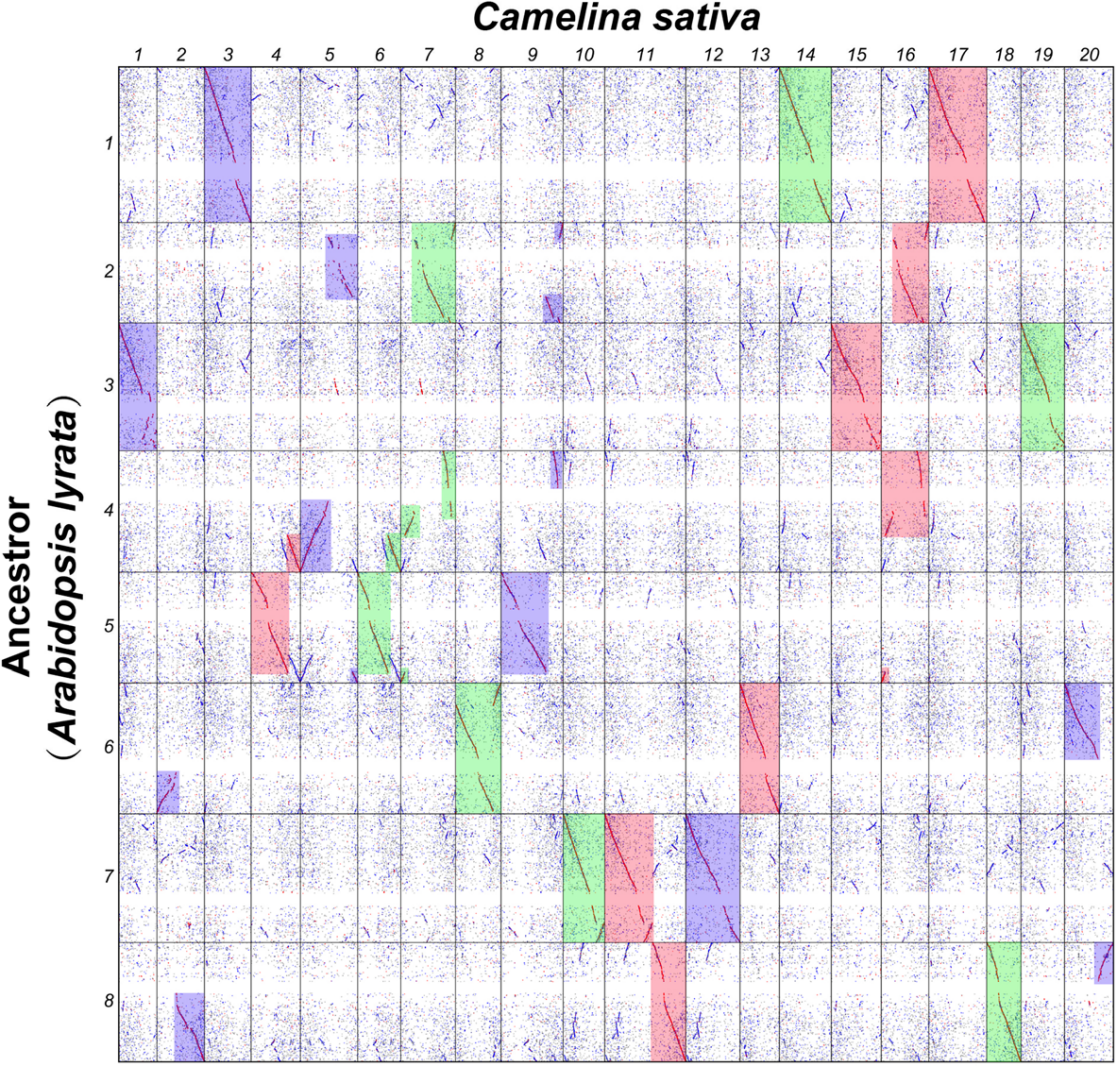
Dot-plot of homologous genes comparing *A. lyrata* and *C. sativa* genomes. *C. sativa* genome is divided into three sub-genomes which are filled in with red (Cs-G1), green (Cs-G2) and blue (Cs-G3) according to syntenic regions. Adopted and modified from Kagale at al. (2014)

Belonging to lineage I of Brassicaceae [3] (Fig. 1), *C. sativa* (false flax) is a high-quality oilseed crop with several advantages of high production and resistance to drought and diseases for industrial production of biodiesel [15]. It was proposed that *C. sativa* represented a whole-genome triplication event relative to *A. thaliana* [16], and three sub-genomes were defined (Cs-G1, Cs-G2 and Cs-G3) [17] (Fig. 2). The sub-genomes Cs-G1and Cs-G2 are more closely related to each other than any of the diploids assayed based on phylogenetic relationship, Cs-G3 shows a clear expression level advantage over the other two sub-genomes, and the three sub-genomes have an almost identical Ks distribution of synteny genes with *A. thaliana*. The three sub-genomes were likely diverged from a common ancestor and the extant *C. sativa* hexaploidy genome result from a two-stage allopolyploid pathway [17]. The genus *Camelina* contains approximate 6 species, including *C. sativa* (2n = 6x = 40), *Camelina microcarpa* (2n = 12, 2n = 4x = 26, 2n = 6x = 40) [18], *Camelina hispida* (2n = 2x = 14), *Camelina rumelica* (2n = 4x = 26), *Camelina neglecta* (2n = 2x = 12) [19], *Camelina laxa* [20] (2n = 2x = 12). By phylogenetic analyses of a set of unanchored genome scaffolds, it was proposed that one *C. microcarpa* accession (2n = 26) included the two sub-genomes of *C. sativa* (Cs-G1 and Cs-G2), showing that the *C. microcarpa* may be the crop’s wild ancestor. The third sub-genome shares significant homology to *C. hispida* (2n = 14), implying this may represent an extant progenitor of the sub-genome (Cs-G3) [21].

The ancestral diploid karyotype of *C. sativa* (derivative of ACK, dACK) were reconstructed based on synteny and colinearity between *C. sativa* and Arabidopsis species [17]. However, dynamic changes of their formation or evolutionary trajectories of extant *C. sativa* chromosomes have not been well inferred. Here, using the theory of telomere-centric genome repatterning, we inferred a different ancestral diploid karyotype of *C. sativa* (ADK), and compared to previous inference, and reconstructed the ancestral karyotypes and evolutionary trajectories of the extant *C. sativa* genome. The present work will contribute to understanding the formation and evolution of the chromosomes in *C. sativa* and other Brassicaceae plants.

## Results

### Inference of ancestral diploid karyotype of *C. sativa*

**Fig. 3:**
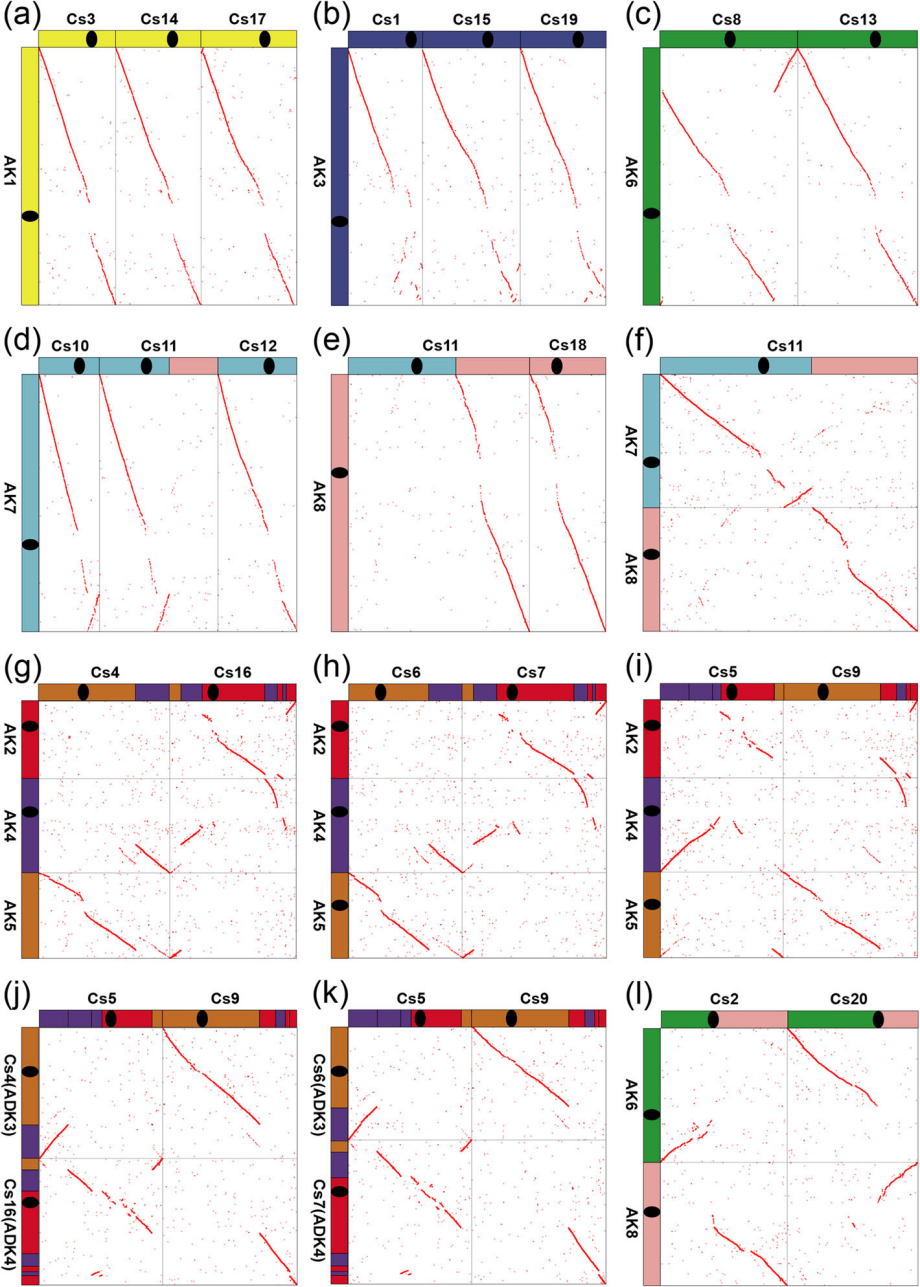
Homologous dot-plots between selected *A. lyrata* or *C. sativa* and *C. sativa* chromosomes. Cs, *C. sativa*. **a** formation of Cs3, 14, 17; **b** formation of Cs1, 15, 19; **c** formation of Cs8, 13; **d** formation of Cs10, 12; **e** formation of Cs18; **f** formation of Cs11; **g** formation of Cs4, 16; **h** formation of Cs6, 7; **i**, **j** and **k** formation of Cs5, 9; **l** formation of Cs2, 20

To understand the evolutionary trajectories of ADK before divergence of three *C. sativa* sub-genomes, we analyzed the syntenic conservation and chromosome repatterning between the genomes of the ancestor of lineage I and *C. sativa*. Here, we took the *A. lyrata* genome as the reference of ancestral genome of lineage I for the sake of the significant colinearity between their genomes (Fig. S1 and Table S1) and high similarity between their karyotype. By searching homologous genes between them, we drew homologous gene dot-plots (Figs. 2 and 3), and showed orthologous correspondence between ancestral genomes of lineage I and *C. sativa* genomes.

In the homologous gene dot-plots of the two genomes, produced directly by using BLASTP hits and further highlighted by integrating inferred colinear genes, every chromosome in the ancestral genome has three homoeologous chromosomes or groups of homoeologous chromosome regions in *C. sativa* genome. We found that 5 ACK chromosomes had nearly perfect orthologous correspondence with at least one or more complete chromosomes in *C. sativa* (Fig. 3a, b, c, d, and e), showing that the integrity of each of these 5 chromosomes in ADK (correspondingly defined as ADK chromosomes 1, 2, 5, 6, 7), which directly inherited the chromosome structure of ACK (AK chromosomes 1, 3, 6, 7, 8) without prominent DNA rearrangements.

**Fig. 4:**
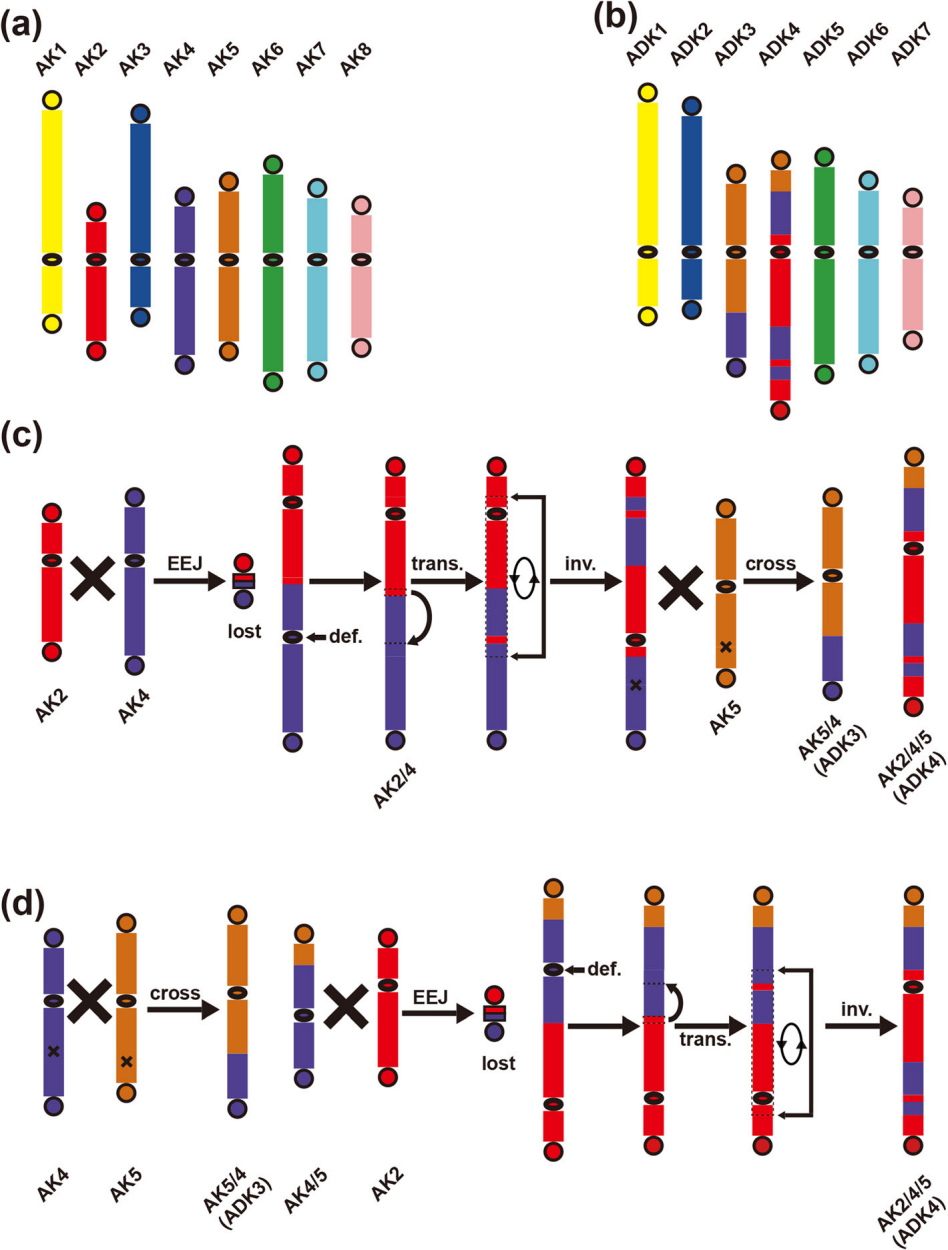
Evolutionary trajectories from ACK to ADK. **a** Ancestral Crucifer Karyotype (ACK). **b** Ancestral Diploid Karyotype of *C. sativa* (ADK). **c**, **d** two comparative evolutionary trajectories of chromosomes ADK3, 4

Notably, orthologous correspondence between AK2, 4, 5 and Cs4, 16 (Cs-G1) is nearly the same as that between AK2, 4, 5 and Cs6, 7 (Cs-G2) (Fig. 3g and h), indicating that Cs-G1 and Cs-G2 shared two ancestral chromosomes, which majorly formed through reciprocal translocation of arms (RTA) and end-end joining (EEJ) between AK2, 4, 5. By searching shared gene synteny between *A. lyrata* and *C. sativa* genomes, we further found that the crossing-over positions between chromosomes (AK4, 5) were respectively between gene AL482377 (Corresponding *C. sativa* ortholog: Csa16g006880.1) and AL321151 (Csa04g046610.1) in AK4, and that between gene AL486375 (Csa04g046590.1) and AL486377 (Csa16g006870.1) in AK5. Actually, the following two evolutionary trajectories could explain the changes of these chromosomes. A relatively more complex evolutionary trajectory could occur as follows: AK2 and AK4 crossed over near one telomere of each of them, resulting in EEJ to produce AK2/4 and formation of a satellite chromosome of two telomeres (and possibly little DNA); then cross-over between AK5 and neo-AK2/4, which experienced one extra translocation and pericentric inversion, resulting in RTA between the two chromosomes to produce AK5/4 (ADK3) and ADK2/4/5 (ADK4) (Fig. 4c). An alternative trajectory could occur as follows: a cross-over between AK4 and AK5 resulted in reciprocal translocation of arms (RTA) to produce AK5/4, forming ADK3, and intermediate AK4/5. Then, AK4/5 and AK2 crossed over near one telomere of each of them, resulting in chromosome end–end joining (EEJ) to produce AK2/4/5 and likely formation of a satellite chromosome by two telomeres (and possibly little DNA). The neo-chromosome AK2/4/5 experienced one extra translocation and pericentric inversion to form ADK4 (Fig. 4d). No matter which trajectory was the actual one, the satellite chromosome likely produced was lost, eventually reducing the chromosome number from 8 in ACK to 7 in ADK.

**Fig. 5:**
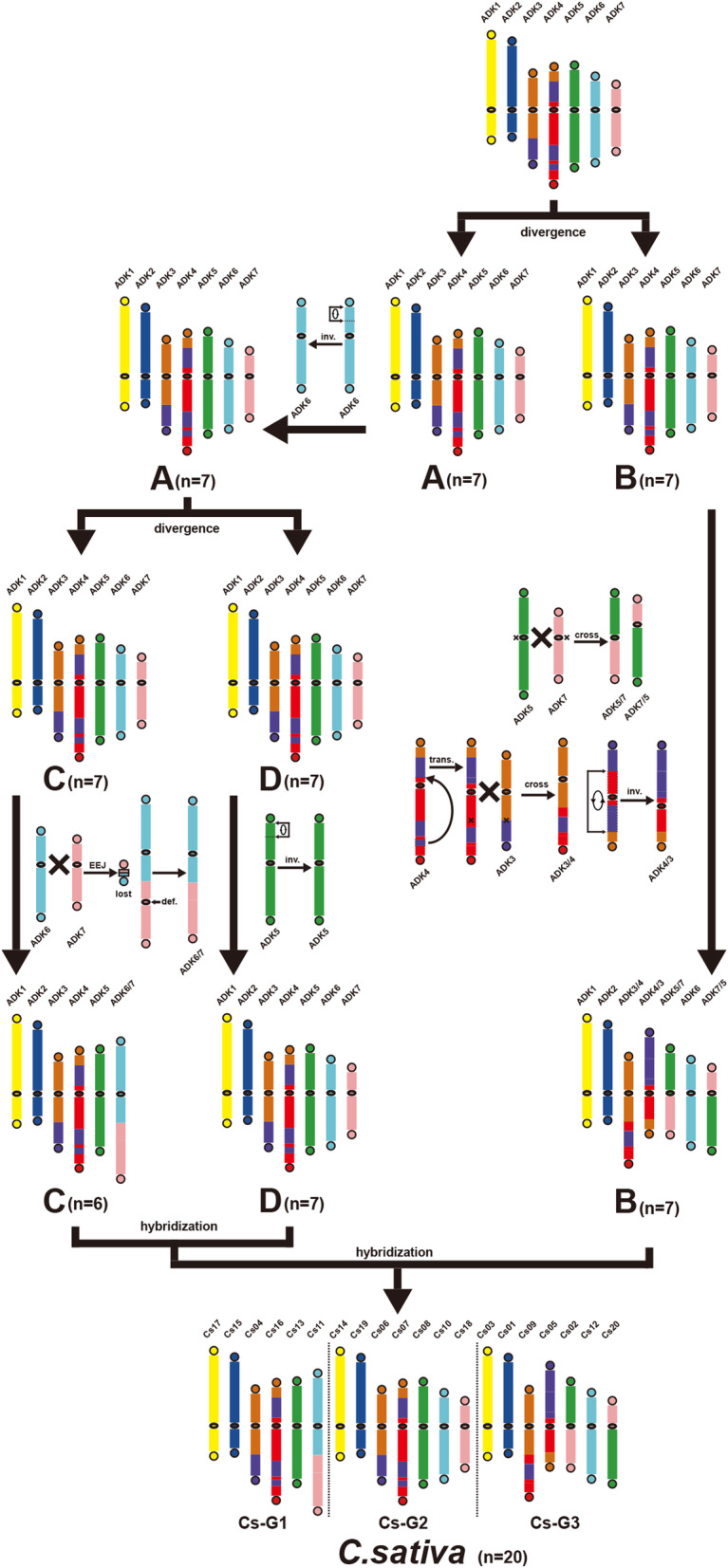
Evolutionary trajectories from ADK to extant *C. sativa* karyotype

Orthologous correspondence between AK2, 4, 5 and Cs5, 9 (Cs-G3) (Fig. 3i) is much different from that between AK2, 4, 5 and Cs4, 16 (Cs-G1) or Cs6, 7 (Cs-G2), showing that Cs5, 9 has particular structures not shared with the other two sets of chromosomes (Cs4, 16 and Cs6, 7). It seems that Cs-G3 does not share the two ancestral chromosomes (ADK3, 4) with Cs-G1 and Cs-G2. However, orthologous correspondence between Cs4, 16 or Cs6, 7 and Cs5, 9 (Fig. 3j and k), showing that Cs5, 9 are majorly formed by RTA between ADK3 and ADK 4 (Fig. 5). By searching gene synteny between *A. lyrata* and *C. sativa* genomes, we further characterized the crossing-over positions between chromosomes (ADK3, 4) are respectively between gene AL486375 (Corresponding *C. sativa* ortholog: Csa04g046590.1) and AL321151 (Csa04g046610.1) in ADK3 (where chromosome arms of AK4, 5 combined), and that between gene AL476152 (Csa09g071500.1) and AL926342 (Csa09g071510.1) in ADK4. This findings provide a clear evidence to support that the Cs-G3 actually inherited karyotype structures of the two ancestral chromosomes (ADK3, 4), which are shared with Cs-G1 and Cs-G2.

### Inferring evolutionary trajectories from ADK to extant *C. sativa* karyotype

Shared chromosome structural patterns can help understand phylogenomic relationship. In homologous gene dot-plots, orthologous correspondence between AK7 (ADK6) and Cs10, 11, 12 (Fig. 3d) suggested that one paracentric inversion is common to Cs10 and Cs11, respectively corresponding to Cs-G1 and Cs-G2, respectively, but not in chromosome Cs12 from Cs-G3. It suggested that Cs-G1 and Cs-G2 are not directly diverged from ADK, but share a common ancestor with one paracentric inversion as compared to ADK6.

The formation process of the three sub-genomes and *C. sativa* genomes could occur as follows: the ancestral diploid of *C. sativa* differentiated into species A and B firstly, and then species A differentiated into species C and D after one paracentric inversion occurred in ADK6 (Fig. 5). Crossing-over between ADK6 and ADK7 occurred near one telomere of each chromosome in species C, resulting in chromosome end–end joining (EEJ) to produce ADK6/7 and formation of a satellite chromosome of two telomeres and little DNA. ADK5 in species D experienced one paracentric inversion independently (Fig. 3c and 5). Crossing-over occurred between ADK3 and ADK4 in species B, which experienced one translocation, resulting in reciprocal translocation of arms (RTA) to produce ADK3/4 and ADK4/3, which experienced one pericentric inversion (Fig. 3j and 5). RTA between ADK5 and ADK7 in species B occurred to produce ADK5/7 and ADK7/5 (Fig. 3j and 5). The crossing-over positions between chromosomes (ADK5, 7) are respectively between gene AL489681 (Corresponding *C. sativa* ortholog: Csa20g058860.1) and AL351869 (Csa02g002270.1) in ADK5 (the region where the centromere of ADK5 is located), and that between gene AL494932 (Csa20g041660.1) and AL494934 (Csa02g033470.1) in ADK7 (the region where the centromere of ADK7 is located). An initial hybridization event between species C (Cs-G1) and D (Cs-G2), resulting in a tetraploid genome, followed by an additional hybridization event between the tetraploid genome and species B (Cs-G3), eventually forming the extant hexaploid genome of *C. sativa* [17] (Fig. 5).

During the formation of the karyotype of *C. sativa*, 14 chromosomes of *C. sativa* inherited the chromosome structures of ADK ones. While one paracentric inversion occurred in Cs-G2 to produce one new chromosome, two RTAs occurred in Cs-G3 with one translocation and pericentric inversion to produce four new chromosomes. EEJ occurred in Cs-G1 to produce one new chromosome and one satellite chromosome. The loss of the satellite chromosomes resulted in the chromosome number reduction from 21 to 20.

## Discussion

A telomere-centric theory shows the likely karyotype changes likely involve the production of free-end chromosomes, which were eventually inserted into other chromosomes, and/or the end-ending joining of different chromosomes [12, 13]. Actually, chromosome may form a circular form and cross-over may occur near its two telomeres, and the resolution of the cross-over may produce a telomere-free chromosome and a satellite chromosome of two telomeres and little DNA; the telomere-free chromosome may invade another chromosome and eventually result in the merge of the invading one into the invaded one, referred to nested chromosome fusion (NCF). Alternatively, two chromosomes may cross over near one telomere of each chromosome, resulting in chromosome end–end joining (EEJ) and formation of a satellite chromosome. Besides, reciprocal translocation of arms (RTA) may although occur. The loss of satellite chromosome explains chromosome number reduction. Here, we used the telomere-centric model to update the explanation of the Brassicaceae karyotype evolution.

Though karyotypes that we inferred are of the same chromosome numbers in key evolutionary nodes as in previous [17], the karyotypes or the chromosome formations are updated. The ancestral diploid karyotype of *C. sativa*, inferred by previous study, only involved chromosome correspondence between AK2 and AK4, but ignores that AK5 should have also taken part in the formation of the ADK3 and ADK4, which is strongly suggested in homologous gene dot-plots between *A. lyrata* and *C. sativa* genomes (Fig. 3j, h; Fig. 4). Comparing to previous study [17], we further inferred the evolutionary trajectories from ADK to extant *C. sativa* karyotype, which involved one EEJ and two RTAs. Previously, the three sub-genomes of *C. sativa* were regarded as having no genome fractionation bias [17]. However, the sharing two ancestral chromosomes by Cs-G1 and Cs-G2, having evolved from ADK chromosomes, provided clear evidences to show their higher similarity and less fractionation as compared to the other chromosome.

Chromosome number reduction (CNR) in Brassicaceae plants took always the end-end-joining or EEJ mechanism rather than the nested-chromosome fusion or NCF mechanism. NCF and EEJ, which can generate satellite chromosome(s), the loss of which resulted in the CNR. Interestingly, the occurrence of the two mechanisms of CNR always showed an obvious plant family preference. The number of occurrences of the two mechanisms in grass family is summerized as follows: from the common 12 ancestral chromosomes, 7 NCFs and 0 EEJ occurred to produce 5 extant Brachypodium chromosomes, 5 NCFs and 1 EEJ to form wheat chromosomes, 1 NCF and 0 EEJ to form foxtail millet chromosomes, 13 NCFs and 4 EEJs to form maize chromosomes. In summary, there are 23 NCFs and 5 EEJs occurring independently to form extant grass chromosomes, showing NCFs were significantly more preferred than EEJ (Chisq-test *P*-value ≈ 0.02395). In contrast, the CNR during the formation of *A. thaliana* chromosomes from eight ancestral chromosomes involved only three EEJs but not NCF [12]. Similar to *A. thaliana*, the formation of ADK, and the formation of the extant hexaploid genome of *C. sativa*, EEJ is the only mechanism that causes CNR. This shows an exclusive preference of EEJ in Brassicaceae. A significant preference of EEJ over NCF was also observed in legumes. Though the sampled families are still too limited, it seems that eudicots prefer EEJ, and monocots prefer NCF, resulting in CNR.

While homologous gene dot-plots are always used to infer gene colinearity in a genome or between genomes, and multiple layers of gene colinearity would suggest the occurrence and ploid levels of polyploidization [10, 22]. Using the assistance of homologous gene dot-plots, it was shown that the cucurbits shared a tetraploid ancestor overlooked by multiple genome sequencing efforts [22], highlighting its unelectable values in genome structure analysis. Besides, homologous gene dot-plots can also intuitively show chromosome changes and trace of genome repatterning [12–14]. A recent effort characterized gene colinearity between more than ten legumes and reconstructed the karyotypes of ancestral nodes during the divergence of legumes and evolutionary trajectories of legume chromosomes [14]. Here, we exploited the gene colinearity patterns in homologous dot-plots and inferred karyotype evolution and even phylogenetic relationship in Brassicaceae plants, further consolidating its usage in ancestral karyotype inference.

## Conclusions

By using the telomere-centric model, we inferred ancestral diploid karyotype of *C. sativa* (ADK), including 7 ancestral chromosomes, and reconstructed the karyotype evolutionary trajectories leading to the formation of *C. sativa* genome. The process involved 2 chromosome fusions. By the analysis of chromosome structure and karyotype evolution, we found that sub-genomes Cs-G1 and Cs-G2 may share a closer common ancestor than Cs-G3. The present work will contribute to understanding the formation and evolution of the chromosomes in *C. sativa* and other Brassicaceae plants.

## Methods

### Plant genome data sets

The genomes of *A. lyrata* [23] and *C. sativa* [17] were downloaded from NCBI (https://ftp.ncbi.nlm.nih.gov/genomes/all/GCF/000/004/255/GCF_000004255.2_v.1.0, https://ftp.ncbi.nlm.nih.gov/genomes/all/GCF/000/633/955/GCF_000633955.1_Cs).

### Dot-plot generation

We used BLASTP [24] to search for homologous pairs (E-value < 1 × 10^− 5^) between every possible pair of chromosomes in two genomes. The best, second best, and other matches with E-value >1e-5 were displayed in different colors, to help distinguish orthology from paralogy, or layers of paralogy as a result of recursive WGD events. Dot-plots were produced using home-made Python scripts.

### Circos diagram generate and inferring positions of breakpoints

Homologous pairs detected by BLASTP were used as input for ColinearScan 1.01 [25] to obtain syntenic regions between *A. lyrata* and *C. sativa* genomes. The maximum gap length (mg) was set to be 50 intervening genes between neighboring genes in colinearity on both chromosomes. Circos diagram of the two genome were produced by TBtools [26] based on the colinearity. Searching the syntenic regions which were involved in RTA to find out the boundary of these regions. The positions of breakpoints were between the boundary of the two syntenic regions.

## Supplementary information

**Additional file 1: Fig. S1** Circos diagram of the colinearity between *A. lyrata* and *C. sativa* genomes. Al, *A. lyrata*; Cs, *C. sativa*. *A. lyrata* and *C. sativa* chromosomes are respectively marked in red and green. The syntenic regions which are connected with different Al chromosmes are shown in different colors.

**Additional file 2: Table S1** Evaluation of homologous regions between Al and Cs genomes.

## Data Availability

The genomes of *A. lyrata* and *C. sativa* were downloaded from NCBI (https://ftp.ncbi.nlm.nih.gov/genomes/all/GCF/000/004/255/GCF_000004255.2_v.1.0, https://ftp.ncbi.nlm.nih.gov/genomes/all/GCF/000/633/955/GCF_000633955.1_Cs) and the raw data supporting the conclusions of this article will be made available by the authors, without undue reservation, to any qualified researcher.

## Abbreviations

AK: Ancestral karyotype
ACK: Ancestral crucifer karyotype
PCK: Proto-Calepineae karyotype
tPCK: Translocation Proto-Calepineae Karyotype
BCD: Brassicaceae-common duplications
CNR: Chromosome number reduction
NCF: Nested chromosome fusion
EEJ: End–end joining
RTA: Reciprocal translocation of arms
ADK: Ancestral diploid karyotype of *C. sativa*
