# Supplementary figures and images for: An updated explanation of ancestral karyotype changes and reconstruction of evolutionary trajectories to form Camelina sativa chromosomes
Source: BMC Genomics. 2020 Oct 12;21:705. doi: 10.1186/s12864-020-07081-0 (PMC7549213; doi:10.1186/s12864-020-07081-0)

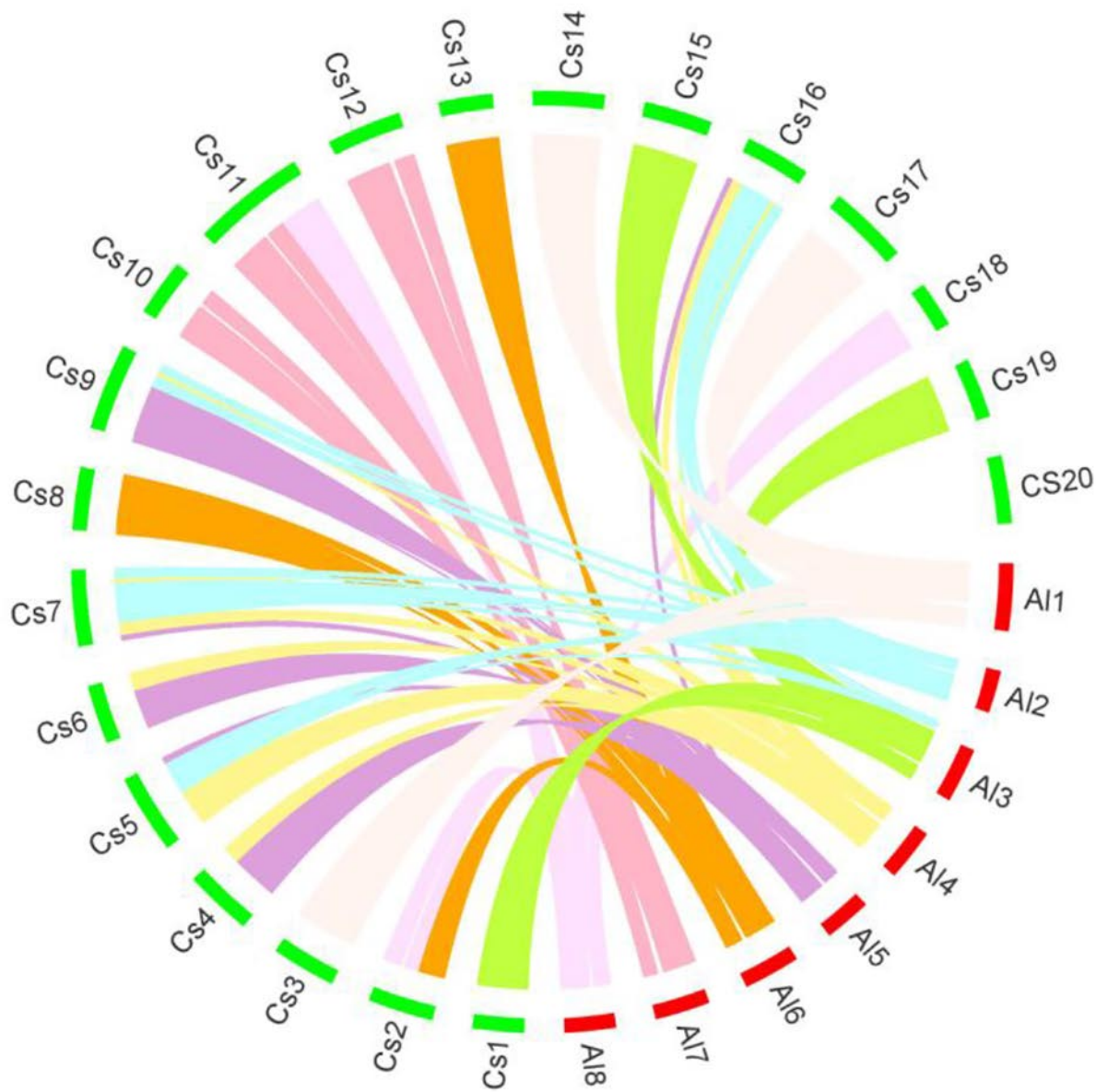

Supplement: Supplementary file 1 — Additional file 1: Fig. S1 Circos diagram of the colinearity between A. lyrata and C. sativa genomes. Al, A. lyrata; Cs, C. sativa. A. lyrata and C. sativa chromosomes are respectively marked in red and green. The syntenic regions which are connected with different Al chromosmes are shown in different colors. [file 12864_2020_7081_MOESM1_ESM.pdf]
